# Chronic Pelvic Pain Prevalence Increases Across Categories of Temporomandibular Disorder Severity in Women: A Large Cross-Sectional Study

**DOI:** 10.1080/24740527.2026.2703519

**Published:** 2026-10-01

**Authors:** Letícia Mignoni, Karin Luana Jung, Maite Prado Matiossi, Mariana Della Valentina Pessoa, Thaís Petrocelli Gonçalves, Luis Fernando Ferreira, Gabriela Tomedi Leites, Patrícia Viana da Rosa

**Affiliations:** a Rehabilitation Sciences (PPG-CR), Federal University of Health Sciences of Porto Alegre (UFCSPA), Porto Alegre, Brazil; b Department of Physical Therapy, Hospital de Pronto Socorro de Porto Alegre (HPS), Porto Alegre, Brazil; c Department of Physical Therapy, Federal University of Health Sciences of Porto Alegre (UFCSPA), Porto Alegre, Brazil; d Teaching and Research Department, Santa Casa of Porto Alegre Hospital, Porto Alegre, Brazil; e School of Electronics, Electrical Engineering and Computer Science, Queen’s University Belfast, Belfast, Northern Ireland, UK

**Keywords:** Temporomandibular disorders, chronic pelvic pain, women’s health, musculoskeletal pain

## Abstract

**Objectives:**

Temporomandibular disorders (TMD) and chronic pelvic pain (CPP) are recognized as chronic overlapping pain conditions that frequently affect women. However, evidence regarding how CPP prevalence varies across categories of TMD symptom severity remains limited. This study investigated whether greater TMD symptom severity was associated with a higher prevalence of CPP in adult women.

**Methods:**

This cross-sectional study included 790 women aged 18–65 years. TMD symptom severity was assessed using the Fonseca Anamnestic Index, and CPP was defined as selfreported pelvic pain lasting at least six months. Anxiety symptoms and physical activity were evaluated using the Beck Anxiety Inventory and the International Physical Activity Questionnaire. Sociodemographic and clinical variables were also collected. Group comparisons were performed using chi-square and ANOVA tests, and relative risks (RR) with 95% confidence intervals were estimated.

**Results:**

Greater TMD symptom severity was associated with a higher prevalence of CPP (p < .001). Compared with women without TMD, those with mild TMD had a higher prevalence of CPP (RR = 2.05, 95% CI 1.21–3.48), whereas women with moderate and severe TMD showed nearly fourfold higher prevalence (RR = 3.81, 95% CI 2.30–6.32 and RR = 4.00, 95% CI 2.37–6.75, respectively). Women presenting both conditions also showed a higher prevalence of anxiety symptoms, urinary complaints, and medical comorbidities.

**Discussion:**

Higher TMD symptom severity was associated with a higher prevalence of CPP in adult women, supporting the presence of overlapping pain conditions. These findings reinforce the importance of integrated and multidimensional approaches in the assessment of women with chronic pain.

## RÉSUMÉ

**Objectifs :** Les troubles temporo-mandibulaires et les douleurs pelviennes chroniques sont reconnus comme des affections douloureuses chroniques concomitantes qui touchent fréquemment les femmes. Cependant, les données sur la variation de la prévalence des douleurs pelviennes chroniques selon les catégories de gravité des symptômes de troubles temporo-mandibulaires demeurent limitées. Cette étude visait à déterminer si une plus grande gravité des symptômes de troubles temporo-mandibulaires était associée à une prévalence plus élevée des douleurs pelviennes chroniques chez les femmes adultes.

**Méthodes :** Cette étude transversale a porté sur 790 femmes âgées de 18 à 65 ans. La gravité des symptômes de troubles temporo-mandibulaires a été évaluée à l’aide de l’indice anamnestique de Fonseca, et la présence de douleurs pelviennes chroniques était définie par une douleur pelvienne autodéclarée persistant depuis au moins six mois. Les symptômes d’anxiété et l’activité physique ont été évalués à l’aide de l’Inventaire d’anxiété de Beck et du Questionnaire international sur l’activité physique. Des variables sociodémographiques et cliniques ont également été recueillies. Les groupes ont été comparés au moyen du test du chi carré et d’analyses de variance, et les risques relatifs (RR) ont été estimés avec leurs intervalles de confiance (IC) à 95 %.

**Résultats :** Une plus grande gravité des symptômes de troubles temporo-mandibulaires était associée à une prévalence plus élevée des douleurs pelviennes chroniques (p < 0,001). Comparativement aux femmes ne présentant pas de troubles temporo-mandibulaires, celles qui présentaient des troubles temporo-mandibulaires légers avaient une prévalence plus élevée des douleurs pelviennes chroniques (RR = 2,05; IC à 95 % : 1,21–3,48). Chez les femmes présentant des troubles temporo-mandibulaires modérés ou graves, la prévalence était près de quatre fois supérieure (RR = 3,81; IC à 95 % : 2,30–6,32 et RR = 4,00; IC à 95 % : 2,37–6,75, respectivement). Les femmes présentant les deux affections avaient également une prévalence plus élevée de symptômes d’anxiété, de symptômes urinaires et de comorbidités.

**Discussion :** Une plus grande gravité des symptômes de troubles temporo-mandibulaires était associée à une prévalence plus élevée de douleurs pelviennes chroniques chez les femmes adultes, ce qui étaye l’existence d’affections douloureuses concomitantes. Ces résultats soulignent l’importance d’adopter des approches intégrées et multidimensionnelles pour évaluer les femmes souffrant de douleurs chroniques.

## Introduction

Chronic pain conditions represent an important public health burden worldwide, particularly among women, in whom multiple persistent pain syndromes frequently coexist.^1^ According to the International Association for the Study of Pain (IASP), chronic pain results from the interaction of biological, psychological, and social factors. Among the most prevalent painful conditions in women are temporomandibular disorders (TMD) and chronic pelvic pain (CPP), both classified as chronic overlapping pain conditions (COPCs), which have been described as sharing clinical features and potentially overlapping mechanisms such as central sensitization (CS), myofascial alterations, and mental health disorders.^1^ Women with persistent pain often seek care across multiple specialties, which may delay recognition of overlapping conditions and contribute to greater symptom burden.^2^ Understanding how chronic pain conditions coexist may support more comprehensive and patient-centered approaches in women’s health care.^2^

The American College of Obstetricians and Gynecologists (ACOG) defines CPP as pain in the anatomical pelvis lasting longer than six months, associated with multiple possible gynecological, urological, gastrointestinal, and musculoskeletal etiologies.^3^ CPP is one of the most common conditions leading women to seek diagnosis and treatment, with substantial healthcare costs, estimated at US$5,264.22 for diagnostic procedures and US$8,937 per patient for in-network treatments.^2^ CPP is a prevalent condition worldwide, with reported prevalence estimates ranging from 6.4% to 25.4% among women, depending on the population studied and diagnostic criteria applied.^1^

Similarly, TMD represent a major cause of non-dental pain in the orofacial region and are characterized by pain and dysfunction involving the temporomandibular joint, masticatory muscles, and related craniofacial structures, often accompanied by limited jaw mobility and referred symptoms to the head and neck.^4–6^ Prevalence estimates of TMD vary considerably across studies according to geographic region, study setting, age range, and diagnostic criteria, with reported prevalence ranging from 4.6% to 31% among adults.^7^ Women are more frequently affected by TMD than men across all age groups.^8^ TMD are considered multifactorial conditions involving a combination of initiating, predisposing, and perpetuating factors, including parafunctional habits, trauma, and psychological factors such as anxiety and depression.^5,9,10^ Persistent and recurrent TMD-related pain may lead to limitations in daily activities and negatively affect the quality of life.^5,9^

Both CPP and TMD have multifactorial etiologies, supported by the biopsychosocial model, in which emotional, environmental, and behavioral factors exert significant influence on their development.^1,3,9^ Stress and anxiety have been identified as variables associated with the persistence of these conditions.^9,11^ Previous studies have suggested an association between TMD, pelvic floor dysfunction, and other chronic pain conditions, supporting the concept of COPCs.^1,12^ However, although the coexistence of these conditions has been described, it remains unclear whether the prevalence of CPP differs according to the severity of TMD symptoms.

Given these considerations, it is pertinent to investigate the relationship between CPP and TMD in women, considering the clinical and social impact of these conditions and the need to better understand patterns of co-occurrence.^1,12^ Therefore, this cross-sectional study aimed to examine whether greater TMD symptom severity was associated with the prevalence of CPP in women, contributing to a better understanding of overlapping pain conditions. We hypothesized that greater TMD symptom severity would be associated with higher CPP prevalence, consistent with patterns described within the COPCs framework.

## Methods

This cross-sectional analytical study was conducted between January 2024 and January 2025 and it is reported according to the Checklist for Reporting Results of Internet E-Surveys (CHERRIES).^13^ Women aged 18 to 65 from across Brazil were recruited through an online survey disseminated on social media platforms and women-focused interest groups. This study involving human participants was approved by the Research Ethics Committee of the Federal University of Health Sciences of Porto Alegre (UFCSPA), under approval number 6.610.776 (CAAE: 76176023.6.0000.5345). Informed consent was obtained from all participants.

Before completing the online survey, participants read and electronically signed an informed consent form describing the study as an investigation of the association between chronic pelvic pain and pain in the temporomandibular region. Although both conditions were mentioned in the study description, the specific study hypothesis regarding the association between TMD severity and CPP prevalence was not disclosed. Participants were recruited regardless of the presence or absence of temporomandibular disorder or chronic pelvic pain symptoms. Therefore, women with TMD, CPP, both conditions, or neither condition were eligible to participate.

Inclusion criteria were women aged 18–65 years, residing in Brazil, and able to provide informed consent. Only fully completed questionnaires that were formally submitted through the electronic survey platform were available for analysis.

Participants were excluded if they were pregnant or breastfeeding, or if they reported a diagnosis of a central nervous system disorder (e.g., epilepsy, multiple sclerosis, CNS tumor), as these conditions may alter pain perception and neural modulation. Additional exclusion criteria were history of spinal trauma or vertebral fracture, current use of orthodontic appliances, or were actively undergoing physical therapy treatment at the time of participation, as these factors could influence musculoskeletal parameters or pain management. Duplicate responses were identified and removed prior to the statistical analysis.

The survey platform only recorded questionnaires after final submission. Therefore, participants who initiated but did not complete the survey were not registered and could not be quantified. Because the survey platform was configured to record responses only after final submission, view rates, participation rates, and completion rates could not be calculated. To facilitate the identification of duplicate participation, several questionnaire fields were mandatory, including e-mail address and demographic information. During data cleaning, responses were reviewed at the individual participant level, with cross-checking of e-mail addresses and demographic characteristics to identify duplicate or inconsistent participant records. Fourteen duplicate responses were identified and removed prior to statistical analysis. The questionnaire also included open-ended questions, which were individually reviewed as part of the data-cleaning process.

Symptoms of TMD and their severity were evaluated using the Fonseca Anamnestic Questionnaire, a widely used screening instrument in Brazil. The Fonseca questionnaire is a screening tool rather than a diagnostic instrument.^14^ The questionnaire consists of ten items with response options of “yes” (10 points), “sometimes” (5 points), or “no” (0 points), resulting in a total score ranging from 0 to 100.^14^ Severity classifications were defined as follows: no TMD (0–15), mild (20–45), moderate (50–65), and severe (70–100).^14,15^

Anxiety symptoms were assessed using the Beck Anxiety Inventory (BAI), which includes 21 items evaluating physical and cognitive symptoms of anxiety over the previous weeks.^16,17^ Total scores range from 0 to 63 and are categorized as: minimal or absent (0–10), mild (11–19), moderate (20–30), and severe (31–63).^16,17^

CPP was identified through self-reported pain localized to the pelvic region and persisting for at least six months, according to the definition proposed by the ACOG.^3^ The purpose of this measure was to identify participants reporting symptoms compatible with CPP rather than to establish a clinical diagnosis. Although the ACOG recommends a systematic, multidimensional, and interdisciplinary assessment for the clinical evaluation of CPP,^3^ such an approach was beyond the scope of this population-based survey. Additional variables collected in the survey included physical activity, sexual function, anxiety symptoms, medical history, medication use, and quality of life. Participants were classified as reporting symptoms compatible with CPP if they answered affirmatively to the question regarding pelvic pain persisting for at least six months. No other questionnaire responses were used to determine CPP status.

Physical activity was assessed using the short version of the International Physical Activity Questionnaire (IPAQ), which classifies individuals as very active, active, irregularly active, or sedentary based on frequency and duration of reported physical activity during the previous week.^18^

Urinary symptoms were investigated through specific questions about the presence of urinary urgency, increased urinary frequency, nocturia, burning sensation during urination, and the sensation of incomplete bladder emptying, according to the International Continence Society guidelines for the clinical characterization of lower urinary tract symptoms.^19^

The questionnaire also included sociodemographic variables (age, education, marital status, and paid occupation) and self-reported clinical data, including the presence of previous illnesses.

After obtaining data using standardized instruments, statistical analysis was performed. Data were analyzed using SPSS software (version 25.0). Descriptive statistics (frequencies, means, and standard deviations) were performed to characterize the sample. For analysis between groups (None, only TMD, Only CPP, TMD + CPP), Chi-square tests and ANOVA with Tukey’s test were applied for multiple comparisons. Normality was verified by the K-S test. The significance level was set at p < .05. Relative risks (RR) and corresponding 95% confidence intervals were calculated to estimate the association between TMD severity categories and CPP prevalence.

BMI had 18.25% missing data due to unavailable self-reported weight and height. Therefore, BMI was analyzed only as a continuous variable using available cases. Approximately 6% of IPAQ responses were excluded because of implausibly high reported activity levels, to reduce potential overestimation bias.

Potential biases include selection bias due to online voluntary recruitment, information bias due to self-reported measures and recall bias in questionnaires such as the IPAQ and BAI. We minimized bias using validated instruments, removing duplicated responses, and excluding inconsistent physical activity data.

## Results

Initially, 804 women participated in the study. Fourteen duplicate responses were identified and excluded, resulting in a final sample of 790 women. Overall, 647 participants (81.9%) were classified as having TMD and 224 (28.4%) reported symptoms compatible with CPP. Among participants with TMD, 45.7% were classified as having mild TMD, 35.9% moderate TMD, and 18.4% severe TMD. Significant differences were observed among groups for age, marital status, body mass index, occupational status, physical activity level, anxiety symptoms, clinical comorbidities, and urinary symptoms, with less favorable clinical profiles observed among women presenting both conditions. Participant characteristics are presented in Table 1.

**Table 1.** Sociodemographic and clinical characteristics of the participants according to temporomandibular disorders (TMD) and chronic pelvic pain (CPP) status.

Variables n (%)	None n = 126 (15.9)	Only TMD n = 440 (55.7)	Only CPP n = 17 (2.2%)	Both n = 207 (26.2)	p-value
**Age (years)^1^**	34 ± 10.7	30.6 ± 10.6	32.2 ± 10	33.1 ±10.2	0.002*
**Marital Status**					>0.001*
Single	68 (54)	315 (71.6)	7 (41.2)	119 (57.5)	
Married	53 (42.1)	107 (24.3)	9 (52.9)	73 (35.3)	
Divorced	5 (4)	18 (4.1)	1 (5.9)	13 (6.3)	
Widowed	0 (0)	0 (0)	0 (0)	2 (1)	
**Ethnicity**					0.852
White	103 (81.7)	370 (84.1)	14 (82.4)	169 (81.6)	
Black/Brown/ Others	23 (18.3)	70 (15.9)	3 (17.6)	38 (18.4)	
**BMI (kg/m^2^)^1^**	24.1 ± 4	25.1 ± 4.8	23.6 ± 5.9	25.9 ± 5.7	0.021*
**Previous medical condition**					
Metabolic disorder	9 (7.1)	47 (10.7)	0 (0)	37 (17.9)	0.005*
Reproductive system disorder	2 (1.6)	14 (3.2)	3 (17.6)	15 (7.2)	0.002*
Pulmonary disease	0 (0)	16 (3.6)	2 (11.8)	13 (6.3)	0.011*
Anxiety	3 (2.4)	14 (3.2)	0 (0)	12 (5.8)	0.242
Rheumatic disease	3 (2.4)	9 (2)	0 (0)	16 (7.7)	0.002*
Hypertensive disease	2 (1.6)	18 (4.1)	1 (5.9)	7 (3.4)	0.555
Gastrointestinal disease	1 (0.8)	13 (3)	0 (0)	4 (1.9)	0.447
Depression	1 (0.8)	9 (2)	0 (0)	6 (2.9)	0.550
Migraine	0 (0)	7 (1.6)	0 (0)	5 (2.4)	0.343
Dermatological disorder	1 (0.8)	7 (1.6)	0 (0)	2 (1)	0.810
Musculoskeletal disorder	0 (0)	4 (0.9)	0 (0)	6 (2.9)	0.082
Mental disorder	0 (0)	8 (1.8)	0 (0)	2 (1)	0.383
Others	3 (2.4)	18 (4.1)	0 (0)	18 (8.7)	0.023*
**Educational Level**					0.091
High school graduate or less	8 (6.3)	34 (7.7)	3 (17.6)	14 (6.8)	
Some college or undergraduate education	26 (20.6)	144 (32.7)	4 (23.5)	54 (26.1)	
Completed undergraduate degree	23 (18.3)	78 (17.7) 1 (5.9)		36 (17.4)	
Some postgraduate education	8 (6.3)	40 (9.1) 1 (5.9)		21 (10.1)	
Completed postgraduate degree	61 (48.4)	144 (32.7) 8 (47.1)		82 (39.6)	
**Paid employment**	102 (81)	296 (67.3) 11 (64.7)		158 (76.3)	0.007*
**Current employment status**					0.022*
Unemployed	21 (16.7)	134 (30.5) 5 (29.4)		42 (20.3)	
Self-employed	44 (34.9)	121 (27.5) 4 (23.5)		78 (37.7)	
Employed	59 (46.8)	182 (41.4) 8 (47.1)		83 (40.1)	
Retired	2 (1.6)	3 (0.7) 0 (0)		4 (1.9)	
**Alcohol consumption**					0.263
Never	22 (17.5)	62 (14.1)		1 (5.9)	44 (21.3)
Once a month or less	40 (31.7)	146 (33.2)		6 (35.3)	65 (31.4)
2–4 times per month	51 (40.5)	187 (42.5)		6 (35.3)	72 (34.8)
At least 2 times per week	13 (10.3)	45 (10.2)		4 (23.5)	26 (12.6)
**Smoking status**					0.059
Yes	4 (3.2)	23 (5.2)	2 (11.8)	14 (6.8)	
No, but I used to smoke	25 (19.8)	118 (26.9)	3 (17.6)	35 (16.9)	
No, I have never smoked	97 (77)	298 (67.9)	12 (70.6)	158 (76.3)	
**Anxiety Level (based on BAI score)**					>0,001*
Minimal anxiety	108 (85.7)	214 (48.6)	8 (47.1)	61 (29.5)	
Mild anxiety	14 (11.1)	130 (29.5)	5 (29.4)	68 (32.9)	
Moderate anxiety	3 (2.4)	59 (13.4)	2 (11.8)	49 (23.7)	
Severe anxiety	1 (0.8)	37 (8.4)	2 (11.8)	29 (14)	
**Activity Level (based on IPAQ score)**					0.005*
Very Active	37 (30.3)	109 (25)	3 (17.6)	50 (24.4)	
Active	45 (36.9)	197 (45.2)	3 (17.6)	86 (42)	
Irregularly active	30 (24.6)	110 (25.2)	6 (35.3)	52 (25.4)	
Sedentary	10 (8.2)	20 (4.6)	5 (29.4)	17 (8.3)	
**Number of urinary** **symptoms**					>0,001*
0	97 (77)	271 (61.6)	8 (47.1)	74 (35.7)	
1	24 (19)	112 (25.5)	6 (35.3)	60 (29)	
2	3 (2.4)	40 (9.1)	1 (5.9)	47 (22.7)	
More than 3	2 (1.6)	17 (3.9)	2 (11.8)	26 (12.6)	

Figure 1 illustrates the significant association between TMD severity and the presence of CPP, with CPP being more prevalent among women with moderate and severe TMD, whereas the absence of CPP was more frequent among women without TMD or with mild TMD (p < .001). Compared with women without TMD, those with mild TMD had a higher prevalence of CPP (RR = 2.06, 95% CI 1.22–3.49), while moderate and severe TMD were associated with even higher prevalence estimates (RR = 3.82, 95% CI 2.31–6.33 and RR = 4.01, 95% CI 2.37–6.76, respectively) (Figure 2).

**Figure 1.**
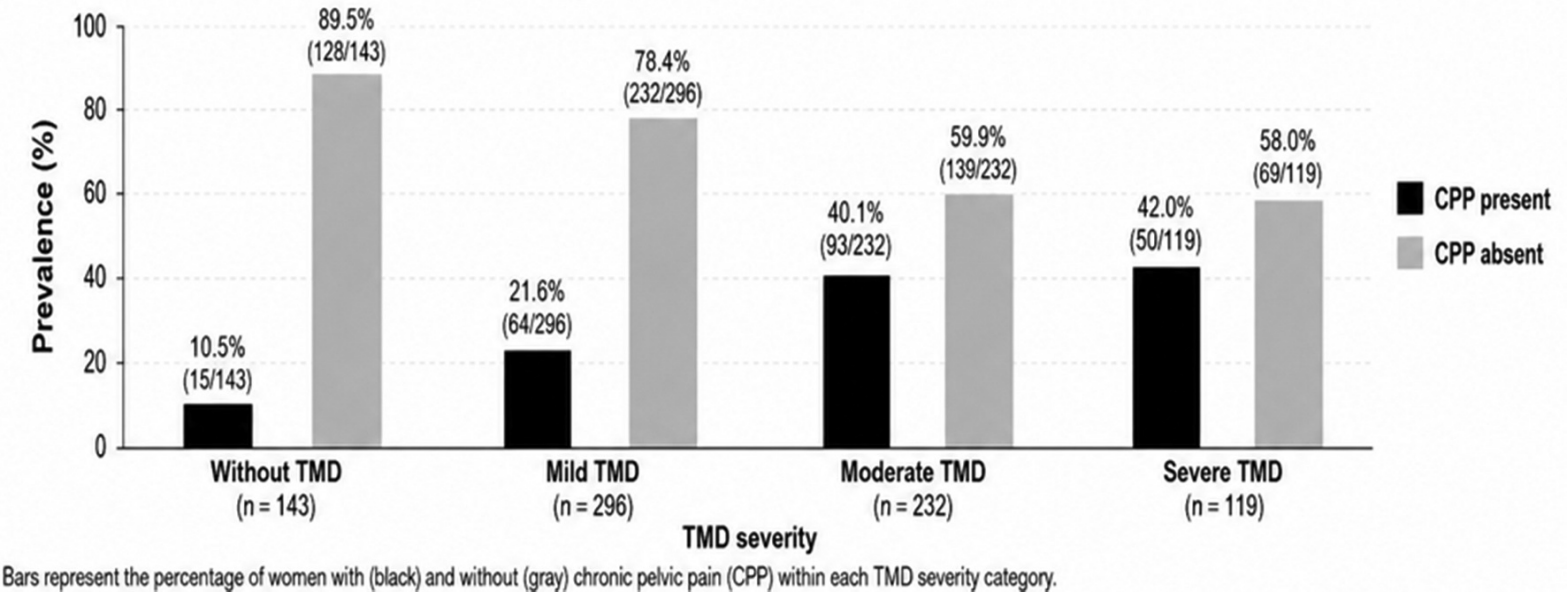
Prevalence of women with and without CPP according to TMD severity.

**Figure 2.**
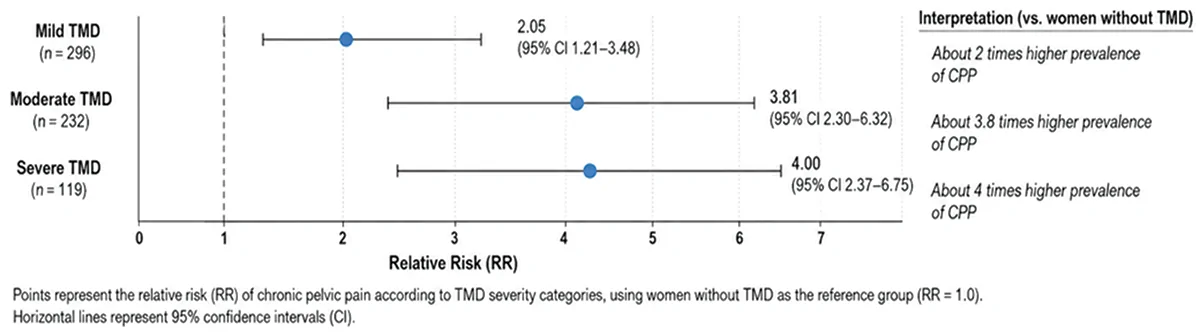
Relative risk of chronic pelvic pain according to TMD severity.

## Discussion

In this large cross-sectional study of adult women, higher levels of TMD symptom severity were associated with higher prevalence of CPP, suggesting a dose–response-like pattern of symptom overlap between these conditions. These findings are consistent with the broader literature on COPCs, in which multiple persistent pain syndromes frequently co-occur.^1,20^

One explanatory model proposed in the literature for the co-occurrence of chronic pain conditions involves altered central pain processing, including features described under CS.^5,21,22^ However, the present study did not directly assess CS or nociplastic pain mechanisms, and therefore, mechanistic interpretations remain hypothetical. A previous study has suggested that altered pain modulation and neuronal convergence may contribute to the coexistence of persistent pain conditions, but these mechanisms cannot be inferred from the current cross-sectional findings.^5,23^ Within this context, our results may reflect a broader overlapping-pain phenotype characterized by greater symptom burden rather than a specific underlying mechanism.^1,21,23^

Beyond the main association, several sociodemographic and clinical characteristics differed across groups, suggesting that women with overlapping TMD and CPP may represent a subgroup with greater overall clinical complexity. Marital status varied across pain categories, although previous evidence suggests that relationship quality may be more relevant than marital status itself in chronic pain outcomes.^8,24^ A higher prevalence of medical comorbidities was observed among women with overlapping TMD and CPP, particularly metabolic, reproductive, pulmonary, and rheumatic conditions. Multimorbidity has been associated with more widespread and intense pain in chronic pain populations, reinforcing its role in amplifying symptom burden.^25^ Notably, rheumatic disorders in our sample included fibromyalgia, lupus, and arthritis, conditions in which nociplastic mechanisms are frequently discussed or may coexist with inflammatory processes.^5,20,22^ Within the broader framework of COPCs, the accumulation of multiple comorbidities may reflect a shared vulnerability to persistent pain rather than condition-specific mechanisms.^1^

Occupational status also differed across groups, with paid employment more frequent among pain-free women and unemployment more common among those with TMD. Socioeconomic determinants, including education and employment, may influence chronic pain risk by shaping access to health information and healthcare services. These findings align with previous evidence linking higher educational attainment to a lower risk of chronic pain.^26^

Anxiety emerged as a distinguishing factor among groups, being more prevalent in women with overlapping TMD and CPP, whereas its absence was more common in pain-free participants. This finding is consistent with previous studies indicating that anxiety frequently co-occurs with chronic pain conditions, including both TMD and CPP.^9,11^ In women with CPP, anxiety symptoms have been associated with greater pain intensity and functional impairment,^11^ and similar associations have been reported in TMD populations.^9^ Although mechanistic pathways were not examined in the present study, psychological distress has been widely described as an important component of the multidimensional experience of chronic pain.^2,11,20^ These findings reinforce the importance of considering psychological factors when evaluating women with persistent pain conditions.

Physical activity levels differed significantly between groups, with sedentary behavior more frequently reported among women with CPP. Previous studies have also described lower physical activity levels in individuals with chronic pain, particularly in women with CPP.^27,28^ Reduced activity may reflect pain-related limitations, fear of symptom exacerbation, or psychological factors commonly observed in chronic pain populations. However, given the cross-sectional design, it is not possible to determine whether sedentary behavior contributes to pain persistence or represents a consequence of ongoing symptoms. These findings suggest that physical activity patterns are an important contextual factor in women with CPP and warrant further investigation in longitudinal studies.

Urinary symptoms were significantly associated with group, being most prevalent in women with both TMD and CPP. In our sample, the accumulation of two or more urinary complaints was particularly frequent in this subgroup, suggesting a greater overall symptom burden among women with overlapping pain conditions. Lower urinary tract symptoms are commonly reported in women with CPP and are often considered part of its broader clinical presentation.^29^ The present findings, therefore, reinforce the observation that CPP frequently coexists with other somatic symptoms, particularly in individuals reporting multiple persistent pain conditions.^1^ However, given the cross-sectional design and the absence of adjustment for all potential confounders, these associations should be interpreted with caution.

From a women’s health care perspective, the coexistence of TMD and CPP may contribute to fragmented clinical pathways, since these conditions are often managed across different specialties. Recognition of overlapping pain symptoms may help clinicians adopt more integrated assessment strategies and improve early identification of women with greater symptom complexity.

The prevalence estimates observed in the present study should be interpreted in the context of the substantial variability reported in the literature. For TMD, prevalence estimates vary considerably according to the population investigated, recruitment methods, and assessment instruments used, ranging from approximately 25.2% to 34.9% in recent systematic reviews and reaching higher estimates in specific populations.^5,7^ Similarly, CPP prevalence has been reported to range from 4% to 43.4% across different populations and study designs,^11^ with worldwide estimates commonly ranging from 6.4% to 25.4% and approximately 15% of women experiencing CPP during their lifetime.^1,2^ Therefore, the prevalence estimates observed in the present study are broadly consistent with the variability reported in previous investigations. Furthermore, the Fonseca Anamnestic Questionnaire used in the present study is a screening instrument designed to assess symptom severity rather than establish a clinical diagnosis of TMD,^14^ while CPP was identified through self-reported symptoms persisting for at least six months according to the ACOG definition.^3^ Consequently, direct comparisons between prevalence estimates across studies should consider differences in case definitions, study populations, and assessment methods.

The cross-sectional design allows identification of associations but does not establish temporal or causal relationships. It, therefore, remains unclear whether TMD precedes CPP, whether CPP precedes TMD, or whether both reflect shared underlying vulnerabilities. The assessment of TMD was based on the Fonseca Anamnestic Index, a screening instrument rather than a diagnostic gold standard,^14^ and no standardized clinical examination was performed to differentiate subtypes. CPP was identified by self report, and differential diagnoses such as endometriosis, interstitial cystitis, or irritable bowel syndrome were not systematically excluded. Also, CPP status was determined through self-report rather than clinical evaluation. Therefore, misclassification cannot be excluded, and the findings should be interpreted as reflecting symptoms compatible with CPP rather than confirmed clinical diagnoses. In addition, because no multivariable adjustment was performed, residual confounding cannot be excluded. Therefore, the observed associations should be interpreted as unadjusted estimates of co-occurrence rather than independent associations.

Another limitation relates to the exclusion criteria adopted in this study. These criteria were established to reduce potential sources of bias and confounding that could influence pain perception, musculoskeletal symptoms, or temporomandibular complaints. However, the exclusion of pregnant and breastfeeding women, individuals with central nervous system disorders, spinal trauma, ongoing orthodontic treatment, or current physical therapy may limit the generalizability of the findings to these populations.

Because recruitment was conducted online through voluntary participation, selection bias cannot be excluded, as participants may differ from the general female population. In addition, pain catastrophizing, direct assessment of central sensitization, and biological markers were not evaluated, limiting the ability to explore potential underlying mechanisms. Future research should incorporate longitudinal designs and more comprehensive clinical and psychophysiological assessments to clarify the temporal relationships and mechanisms underlying the coexistence of TMD and CPP.

An additional limitation concerns the representativeness of the sample. Participants were recruited through a voluntary online survey and, therefore, do not constitute a probabilistic sample of Brazilian women. As commonly observed in web-based health surveys, individuals with greater interest in health-related topics, higher educational attainment, or greater symptom burden may have been more likely to participate. Consequently, the prevalence estimates reported in this study should not be interpreted as nationally representative estimates of TMD or CPP. However, the large sample size and inclusion of participants from different regions of Brazil may have contributed to greater heterogeneity within the study population.

Additionally, although participant-level information was collected and responses, including open-ended responses, were individually reviewed during data cleaning, no specific automated bot-detection mechanism was implemented. Bot-generated responses are an increasingly recognized threat to data integrity in online research, and recent evidence indicates that individual screening strategies may be insufficient to detect increasingly sophisticated automated responses.^30,31^ Although no patterns raising suspicion of automated submissions were identified during data review, the possibility of bot-generated responses cannot be retrospectively and definitively excluded and should, therefore, be considered a potential limitation of the present study.

## Conclusion

This study demonstrated that greater TMD severity is significantly associated with the presence of CPP in adult women. Women with more severe TMD symptoms showed higher prevalence of CPP, reinforcing the frequent coexistence of these conditions. These findings highlight the importance of integrated assessment of overlapping pain conditions in women’s health care. Further longitudinal studies are needed to clarify temporal relationships and underlying mechanisms.

## Data Availability

The dataset generated and analysed during the current study is not publicly available due to ethical and confidentiality restrictions but is available from the corresponding author upon reasonable request.
